# Hair Regeneration Treatment Using Adipose-Derived Stem Cell Conditioned Medium: Follow-up With Trichograms

**Published:** 2015-03-26

**Authors:** Hirotaro Fukuoka, Hirotaka Suga

**Affiliations:** Cherry-Blossom Plastic and Regenerative Surgery, Tokyo, Japan; and Department of Plastic and Reconstructive Surgery, Kyorin University, Tokyo, Japan

**Keywords:** alopecia, adipose-derived stem cells, conditioned medium, trichograms, half-side comparison study

## Abstract

**Objective:** Adipose-derived stem cells secrete various growth factors that promote hair growth. This study examined the effects of adipose-derived stem cell-conditioned medium on alopecia. **Methods:** Adipose-derived stem cell-conditioned medium was intradermally injected in 22 patients (11 men and 11 women) with alopecia. Patients received treatment every 3 to 5 weeks for a total of 6 sessions. Hair numbers were counted using trichograms before and after treatment. A half-side comparison study was also performed in 10 patients (8 men and 2 women). **Results:** Hair numbers were significantly increased after treatment in both male (including those without finasteride administration) and female patients. In the half-side comparison study, the increase in hair numbers was significantly higher on the treatment side than on the placebo side. **Conclusion:** Treatment using adipose-derived stem cell-conditioned medium appears highly effective for alopecia and may represent a new therapy for hair regeneration.

Conservative treatments for alopecia include oral administration of finasteride and topical administration of minoxidil. However, these conventional therapies are ineffective in some patients. Furthermore, finasteride administration is indicated only for androgenic alopecia and is therefore applied only in male patients. New therapies that are both more effective and able to be used in female patients are required.

Adipose-derived stem cells are multipotent cells that have shown potential for regenerative medicine.^1^ Adipose-derived stem cells not only differentiate into mesenchymal lineage cells but also secrete various growth factors.^2^^,^^3^ Recent studies have reported that adipose-derived stem cells promote hair growth via growth factor secretion.^4^^,^^5^ We have already used adipose-derived stem cell-conditioned medium to treat alopecia and reported good results.^6^^,^^7^

In this study, to obtain more objective data, we examined changes in hair numbers as measured using trichograms and evaluated the effects of adipose-derived stem cell-conditioned medium on hair regeneration.

## METHODS

### Patients

The study followed principles in the Declaration of Helsinki. Twenty-two patients (11 men and 11 women; age range, 20-70 years) with alopecia were treated with adipose-derived stem cell-conditioned medium and examined with trichograms both before starting and after completing treatment. In the other group of 10 patients (8 men and 2 women; age range, 20-73 years), a half-side comparison study was performed as described later. Informed consent was obtained from all patients prior to enrolment in the study.

### Treatment

We used a commercial product containing protein solution from adipose-derived stem cell-conditioned medium (AAPE; Prostemics, Seoul, Korea), as previously described.^6^^,^^7^ AAPE® was well studied in the previous report and it contains various growth factors or cytokines such as hepatocyte growth factor, fibroblast growth factor–1, granulocyte colony-stimulating factor, granulocyte macrophage-colony-stimulating factor, interleukin 6, vascular endothelial growth factor, and transforming growth factor β3.^8^ Intradermal injections with a 31-G needle provided about 0.02 mL/cm^2^ of solution. A total volume of 3 to 4 mL was injected during each session of treatment. Patients received treatment every 3 to 5 weeks for a total of 6 sessions. Finasteride was also administered to 6 male patients, but no female patients, during the study.

### Trichograms

The intersection of a line extending cranially from the lateral angle of an eye (left or right) and a line connecting both ears coronally was marked on the scalp with tattoo. A circle 2 cm in diameter centered on this mark was shaved. TrichoScope images were recorded using a PowerShot 450 digital camera (Canon, Tokyo, Japan). The number of hairs within a circle of 11 mm in diameter (area, 95 mm^2^) centered on the tattoo was counted by 2 technicians blind to the patients, and the average number was recorded as data. Trichograms were made before starting treatment and at 1 to 3 months after the final treatment (7 to 12 months after initial treatment).

### Half-side comparison study

After providing informed consent, 10 patients (8 men and 2 women; age range, 20-73 years) received adipose-derived stem cell-conditioned medium treatment on the left side and placebo treatment (saline injection) on the right. Treatment was again performed every 3 to 5 weeks for a total of 6 sessions. Both sides of the scalp were examined using trichograms before starting treatment and after treatment. None of the patients were administered finasteride during this study.

### Statistical analysis

Data are expressed as mean ± standard error of the mean. Data before and after treatment, or data from both sides in the half-side comparison, were compared using the Wilcoxon signed rank test. Values of *P* < .05 were considered statistically significant.

## RESULTS

**Figure 1:**
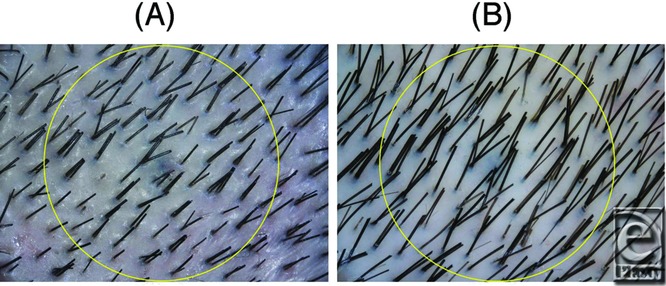
TrichoScope images of a 24-year-old male patient. (A) Before treatment. The number of hairs in the circle is 126. (B) After treatment (7 months after initial treatment). The number of hairs in the circle is 141.

**Figure 2:**
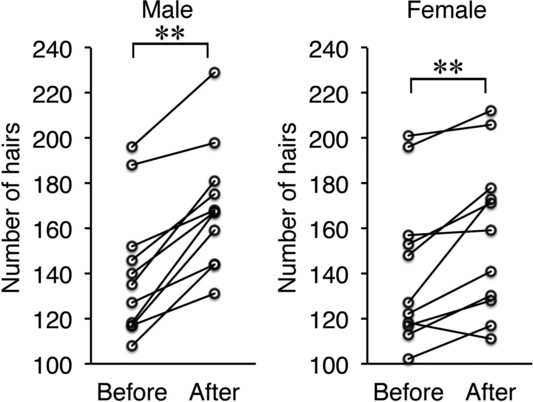
Changes in the number of hairs before and after treatment in male and female patients. *P < .01.

**Figure 3:**
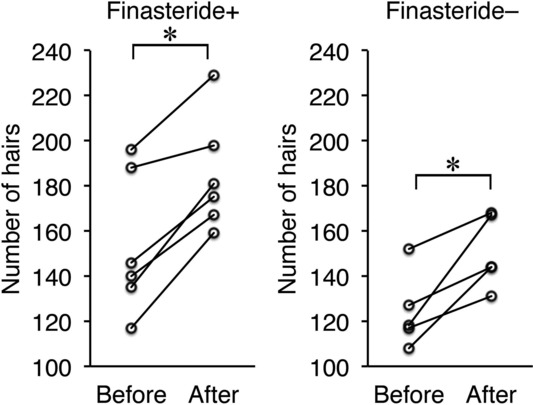
Changes in the number of hairs before and after treatment in male patients with or without finasteride administration. *P < .05.

**Figure 4:**
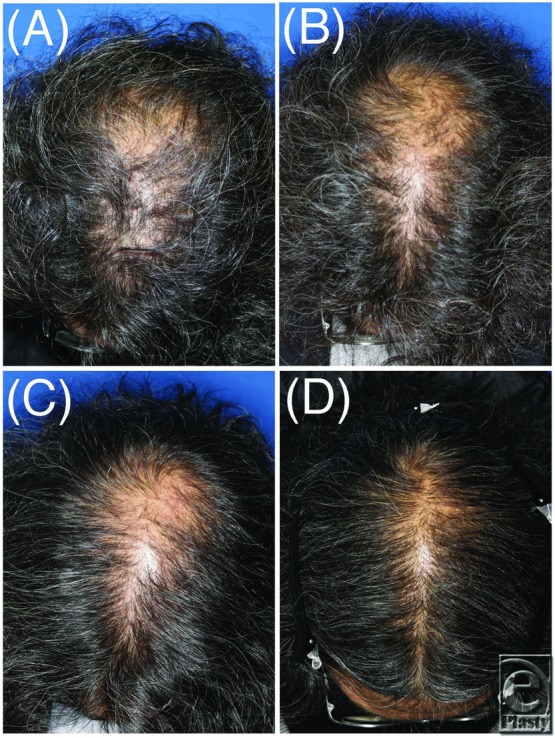
A 49-year-old male patient. (A) Before treatment. (B) During treatment (4 months after initial treatment). (C) After treatment (10 months after initial treatment). (D) Final follow-up (2 years and 1 month after initial treatment).

**Figure 5:**
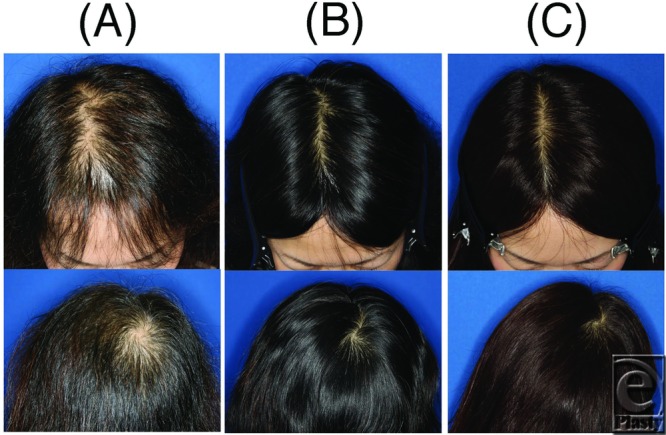
A 44-year-old female patient. (A) Before treatment. (B) After treatment (7 months after initial treatment). (C) Final follow-up (1 year and 10 months after initial treatment).

Numbers of hairs in the same area of the same patient were accurately counted using trichograms before and after treatment (Fig 1). The number of hairs increased significantly after treatment in both male (n = 11) and female (n = 11) patients (Fig 2). The mean increase in the number of hairs was 29 ± 4.1 in male patients and 15.6 ± 4.2 in female patients. No significant difference was observed between men and women. In male patients, groups with (n = 6) and without (n = 5) finasteride administration were compared. The number of hairs increased significantly after treatment in both groups (Fig 3). No significant difference was observed between groups with or without finasteride administration. Representative clinical courses are shown in Figure 4 (a male patient) and Figure 5 (a female patient).

**Figure 6:**
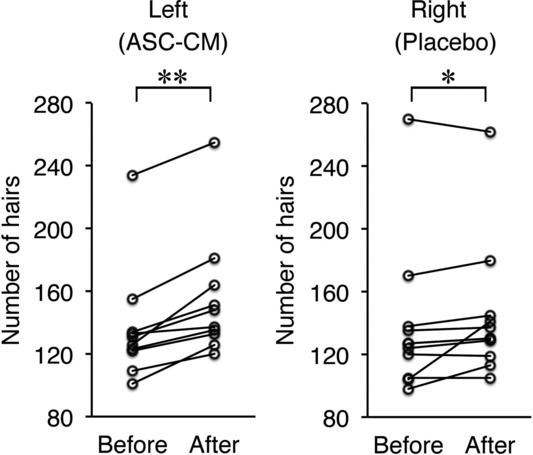
Changes in the number of hairs before and after treatment in the half-side comparison study. *P < .05 and †*P* < .01.

**Figure 7:**
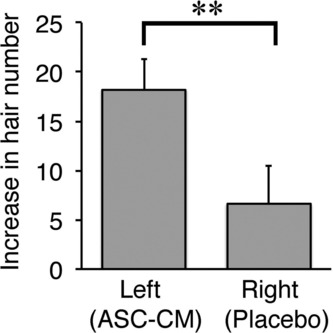
Increases in the number of hairs in the half-side comparison study. *P < .01.

In the half-side comparison study, the number of hairs was significantly increased after treatment on both the left (adipose-derived stem cell-conditioned medium) and right (placebo) sides (Fig 6). However, the increase in the number of hairs was significantly higher on the left (adipose-derived stem cell-conditioned medium) side than on the right (placebo) side (Fig 7).

## DISCUSSION

This study used trichograms to objectively show the positive effects of adipose-derived stem cell-conditioned medium on alopecia. To obtain accurate data, shaving and marking the area for the trichogram is important. We believe that trichograms, particularly as used in this study, offer a reliable and useful method for assessing the treatment of alopecia, although the method is semi-invasive for patients.

Treatment with adipose-derived stem cell-conditioned medium was effective, even in patients who did not take finasteride. We believe that this treatment may offer an alternative to finasteride administration and thus appears particularly useful for female patients. Although no significant difference in improvement of alopecia was seen between male patients treated using adipose-derived stem cell-conditioned medium with and without finasteride administration, we got the impression that the combination of adipose-derived stem cell-conditioned medium and finasteride is preferable to single treatment. The combination therapy was effective in our previous study as well.^6^ We believe that hairs are regulated by various factors, including androgen and cytokines. The efficacy of combination therapy should be elucidated in a future study.

Adipose-derived stem cell-conditioned medium is rich in growth factors such as vascular endothelial growth factor, hepatocyte growth factor, platelet-derived growth factor, and insulin-like growth factor 1.^2^^,^^9^ Vascular endothelial growth factor controls hair growth and follicle size through angiogenesis.^10^ Hepatocyte growth factor is involved in the cyclic growth of hair follicles.^11^^,^^12^ Platelet-derived growth factor induces and maintains the anagen phase of hair follicles.^13^ Insulin-like growth factor 1 controls the hair growth cycle and differentiation of hair shafts.^14^ In treatment with adipose-derived stem cell-conditioned medium, each growth factor seems likely to activate hair follicles and contribute to increasing the number of hairs in patients with alopecia. Because growth factors dose-dependently affect cells, the dose dependence of the effects of adipose-derived stem cell-conditioned medium on alopecia should be examined in a future study.

In the half-side comparison study, the finding that the number of hairs increased even on the right (placebo) side was somewhat surprising. This result might suggest that the injection itself activates hair growth via tissue injury, or that adipose-derived stem cell-conditioned medium injected on one side can affect the other side, perhaps through the local circulation.

Contraindications for adipose-derived stem cell-conditioned medium treatment are similar to those for general mesotherapy, including local skin disease, inflammation, infection, allergic disease, autoimmune disease, pregnancy, cancer, and current anticoagulant therapy. The most common complication is pain during and after injection, but this can be prevented or managed with supraorbital nerve block, occipital nerve block, local anesthesia, cooling, or administration of nonsteroidal anti-inflammatory drugs.

In conclusion, treatment using adipose-derived stem cell-conditioned medium appears highly effective for alopecia and may represent a new avenue of therapy for hair regeneration. More details about adipose-derived stem cell-conditioned medium treatment, such as long-term (over a period of years) effects and histological changes, should be elucidated in the future.
